# Prevalence of physical inactivity and associated factors among older adults in Gondar town, Northwest Ethiopia: a community-based cross-sectional study

**DOI:** 10.1186/s12877-024-04701-2

**Published:** 2024-01-29

**Authors:** Kassaw Belay Shiferaw, Ermias Solomon Yalew, Ashenafi Zemed, Melisew Mekie Yitayal, Gashaw Jember Belay, Melkamu Alie, Alemu Kassaw Kibret, Mihret Dejen Takele, Yohannes Abich, Moges Gashaw

**Affiliations:** Department of Physiotherapy, School of Medicine, College of Medicine and Health Sciences, University of Gondar, Gondar, Ethiopia

**Keywords:** Physical inactivity, Older adults, Prevalence, Factors, Ethiopia

## Abstract

**Introduction:**

Older adults are increasing in number in both developed and developing countries. However, as the world’s aging population grows, the burden of diseases among older people also increases. Despite this, co-occurring health problems due to physical inactivity in older adults have become a concern, and physical inactivity can be caused by different conditions. As a result, for older adults to have better health outcomes, early diagnosis of physical activity status and prevention of non-communicable diseases are crucial. There is a lack of data on the prevalence and associated factors of physical inactivity among older adults that is scarce in sub-Saharan Africa, particularly in the study area, Ethiopia. Therefore, this study aimed to assess the prevalence and associated factors of physical inactivity among older adults in Gondar town, Northwest Ethiopia, in 2022.

**Methods:**

A community-based cross-sectional study was conducted from April 1st to June 30th, 2022, in Gondar town, Northwest Ethiopia. The data was collected by the Global Physical Activity Questionnaire, Volume 2 through face-to-face interviews among 838 participants. Data were entered into Epinfo version 7.1, then exported and analysed using the Statistical Package of Social Science version 25. A binary logistic regression model was performed to identify factors associated with physical inactivity. The result was considered statistically significant based on an adjusted odds ratio of 95% and a *p*-value less than 0.05.

**Result:**

The prevalence of physical inactivity was 65.6% (95% CI: 62.1–68.9). Being female (AOR: 3.053, 95% CI:1.487–6.267), age group > = 80 (AOR: 4.082, 95% CI:1.234–13.497), primary school level (AOR: 3.020, 95% CI:1.433–6.367), no formal education (AOR: 8.573, 95% CI:2.843–25.847), unemployed (AOR: 10.273, 95% CI:5.628–18.753), and symptoms of depression (AOR: 7.152, 95% CI: 4.786–17.965) were significantly associated with physical inactivity.

**Conclusion:**

Physical inactivity was relatively high among older adults in Gondar town. Being female, older age, having low levels of education, being unemployed, and having depression symptoms were associated with physical inactivity. We suggest promoting the health benefits of physical activity among females by reducing their burden, older age groups, and unemployed older adults, and avoiding depression among individuals.

## Background

The global population is increasing in both developed and developing nations, particularly with an increase in the number of older people [1]. However, as the world’s aging population grows, the burden of diseases among older people also increases, posing a problem for global healthcare system, particularly in under developing nations [2]. As people’s lifestyles change around the world, notably with regard to physical inactivity [3]. Older persons should exercise for more than 600 metabolic equivalents (METs) minutes per week, or at least 150 minutes per week of moderate-to-vigorous aerobic activity [4].

Physical inactivity is a serious public health concern worldwide, which is the fourth modifiable risk factor for non-communicable disease mortality and a significant contributor to disability [5]. The consequence of physical inactivity is 3.3 million deaths annually worldwide, of which 6–10% are caused by non-communicable illnesses [6, 7], and the majority of deaths (70%) are caused by physical inactivity in low- and middle-income countries [8]. Physical inactivity is expected to have annual expenditures on the worldwide health-care system of $538 billion [9]. In addition, research on the effects of physical inactivity revealed that it contributed to 10% of breast and colon cancers, 7% of type 2 diabetes mellitus, and 6% of coronary heart ailments [10]. However, the burden of physical inactivity was not well known in Ethiopia.

The prevalence of physical inactivity has increased, it is estimated that 31.1% of people worldwide are physically inactive [11]. Studies reported that the prevalence of physical inactivity among older adults ranged from 5 to 29% in Europe [12], 50.7% in Brazil [13], 63.1% in China [14], 29.8% in Malaysia [15], and 60.5% in South Africa [16]. Additionally, a report revealed that Ethiopia is seeing an increase in the number of people who aren’t physically active [17].

Moreover, different studies revealed that socio-demographic factors (sex, age, educational status, marital status, monthly incomes), body mass index, social support, smoking and alcohol consumption, depression, and history of NCDs were associated with physical inactivity [12, 15, 18–22].

Despite this, co-occurring health problems due to physical inactivity in older adults have become a concern, and the physical inactivity can be caused by different conditions. As a result, for older adults to have better health outcomes, early diagnosis of physical activity status and prevention of NCDs are crucial. Data on prevalence and associated factors of physical inactivity among older adults are scarce in sub-Saharan Africa, particularly in the study area, Ethiopia. Therefore, this study was aimed to assess the prevalence of physical inactivity and associated factors among older adults in Gondar town, Northwest Ethiopia, in 2022.

## Methods

### Study design and setting

A community-based cross-sectional study was conducted from April to June 2022. The study was conducted in Gondar town, Amhara regional state, Northwest Ethiopia. The city is located in the central Gondar zone, Amhara regional state, 748 km northwest of Addis Ababa, Ethiopia’s capital, and about 180 km from Bahir Dar, Amhara regional state’s capital. Gondar is one of the most ancient and largely populated cities in the country. It is at 12˚ 45˚ north latitude and 37˚ 45˚ east longitude, with an elevation of 6,998 feet above sea level. Gondar town has 25 kebeles (the smallest administrative units in Ethiopia). According to the Gondar Statistics Agency’s 2021/22 projection from 2007 population census data, the total population of Gondar town was estimated at 390,000, more than half of the population were women, and more than 9000 were older adults. Despite the population of the city increasing over time, it does not have well-designed and accessible infrastructure for physical activity, such as playgrounds, parks, sidewalks, bike lanes, and good public transportation systems that promote physical activities like walking and cycling, and as a result, it increases physical inactivity rates.

### Source population, study population, inclusion and exclusion criteria

The source population was all older adults living in Gondar town. Older adults aged 60 years and older in selected kebeles during the study period were the study population. Older adults aged 60 years and older who were permanent residents (≥ 6 months) in the selected kebeles were included. However, older adults who were bedridden and unable to communicate during the data collection period were excluded.

### Sample size determination and sampling procedure

The sample size was calculated by using the single population proportions formula, using the assumption of a 95% confidence interval, (α = 5%) level of significance, a marginal error of 5%, a 10% non-response rate, and a 45.5% prevalence of physical inactivity among adults [23].

$$ n = \frac{{\left(z\frac{a}{2}\right)}^{2}p(1-p)}{{d}^{2}} $$

n = sample size.

α = (level of significance) = 5%,

Z = value of z statistic at 95% confidence level,

d = margin of error allowed = 5%,

*P* = 45.5%

$$ \text{n = }\frac{{\left(\text{1.96}\right)}^{\text{2}}\left(\text{0.545}\right)\left(\text{0.455}\right)}{{\left(\text{0.05}\right)}^{\text{2}}}\text{=381}$$

Finally, the final sample size was 838, including two design effects and 10% non-response rate.

**Fig. 1:**
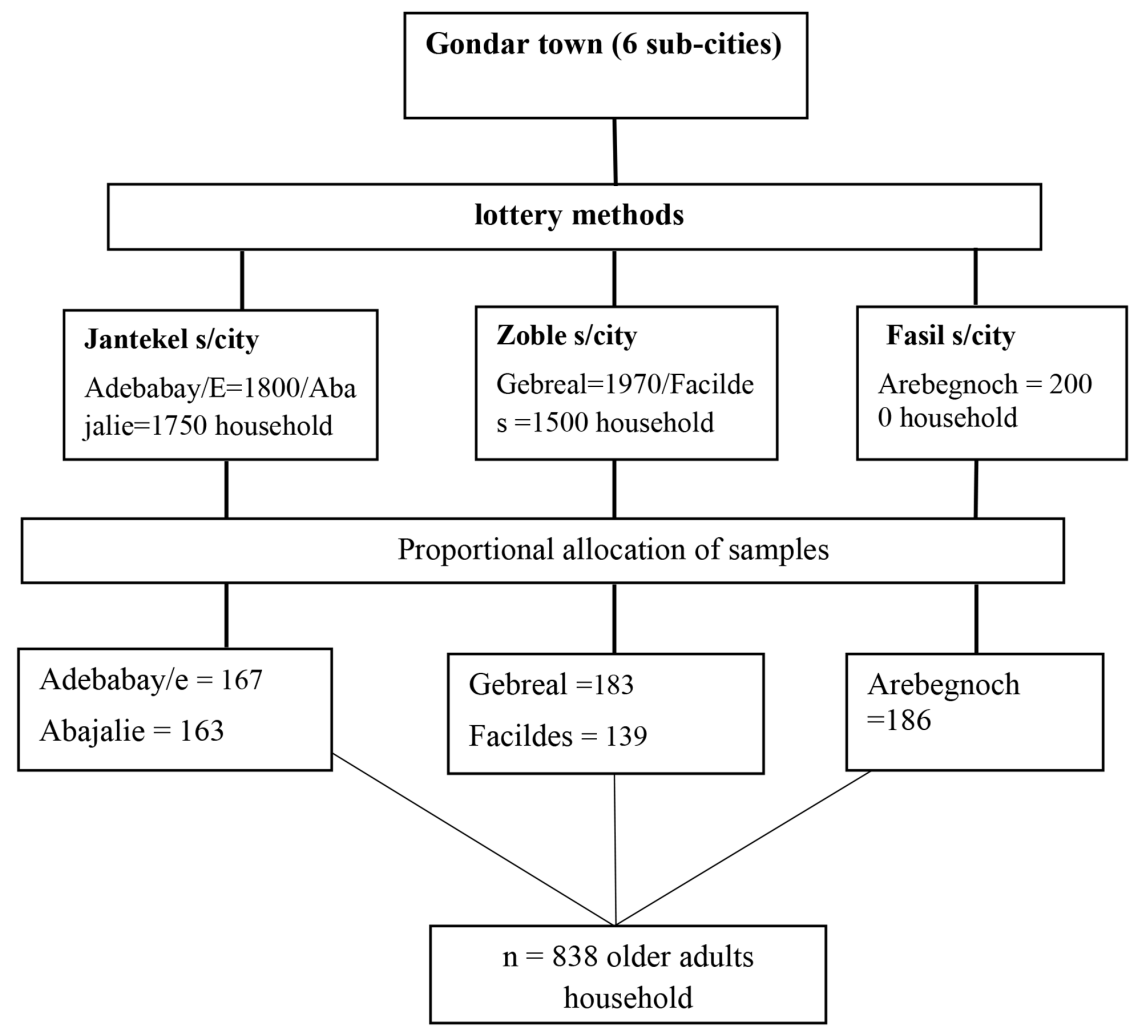
Sampling procedure of older adults at Gondar town, Northwest Ethiopia, 2022

A multi-stage sampling technique was used to select the required sample. In the first step of sampling, three administrative areas (sub-cities), Jantekel, Zoble, and Facil were selected by using a simple random sampling technique from six sub-cities. In the second step of sampling, Adebabay Eyesus and Abajalie Kebeles from Jantekel sub-city, Gebreal and Facildes Kebeles from Zobel sub-city, and Arebegnoch from Facil sub-city were selected randomly. A total of 3,550 households in Abajalie and Adebabay Eyesus, 3,470 households in Gebreal and Facildes, and 2000 households in Arbegnoch Kebeles were found. Then, after proportional allocation to each selected kebeles, a systematic sampling technique was used to select households from each of the kebeles. The interval (𝐾) value was calculated by dividing the total household of the selected kebele by the total calculated sample size. The initial household was randomly selected by lottery method. Then other households were selected at every 11th interval. Whenever more than one eligible older adult was found in the same selected household, only one of them was chosen using the lottery method for interview. In case no eligible candidate was identified in a selected household or the selected household was closed even after revisit, the sampling process continued to the next household in a clockwise direction until getting an eligible person. See sampling procedure in Fig. 1.

### Operational definition

#### Older adults

are defined by the UN as a person aged 60 years or older [24].

#### Physical inactivity

Participants who achieved less than 600 MET-minutes per week total physical activity level were classified as “physically inactive” [25].

#### Depression

is measured by the Geriatrics Depression Scale, which has 15-item questions. A score from 0 to 4 is normal, and a score ≥ 5 suggests depression [26].

#### Social support

is assessed by the OSSS-3. The sum score ranges from 3 to 14, with scores of 3–8 poor social support, 9–11 moderate social support, and 12–14 strong social support [27].

#### Cigarette smoking

Those who smoke any tobacco products daily were considered tobacco users [28].

#### Alcohol drinking

Those who consumed any type of alcohol with 2 or more bottles daily were considered alcohol users [29].

#### Body Mass Index (BMI)

According to the Centres for Disease Control and Prevention, BMI calculated as weight in kilograms divided by height in square meters and interpreted as underweight <(18.5), normal (18.5–24.9), overweight (25.0-29.9), and obese ≥(30.0) [30].

### Data Collection instrument

The Global Physical Activity Questionnaire (GPAQ) version 2 was used to collect the data through face-to-face interviews [31]. During an interview, a GPAQ generic show card was displayed for the study participants to clarify types of physical activity. The reliability and validity of the Global Physical Activity Questionnaire (GPAQ) have been confirmed in many countries [32]. Questionnaires were answered by the older adult population about the frequency and duration of physical activity in the past 7 days to estimate the weekly time spent in PA (min/wk.). Which means, moderate MET minutes/week = 4.0 * moderate minutes * days, vigorous MET-minutes/week = 8.0 * vigorous activity minutes * days, and walking MET-minutes/week = 4 * walking minutes/cycling * days, if the combination of these < 600 MET-minutes/week total physical activity level is classified as *“*physically inactive according to GPAQ analysis guidelines.

Depression was assessed by geriatric depression scale, which is a self-reported scale. It is easy to administer, use, and freely available. It has 15-item questions. Older adults give response to what they feel within the past one week. It is scored as one point for each one selected. A score of 0 to 4 is normal and ≥ 5 suggest depression [26]. Social support was assessed by OSSS-3. The sum score ranges from 3 to 14. Scores of 3–8 poor social support, 9–11 moderate social support, and 12– 14 strong social support [27]. Moreover, weight and height were measured twice. The weight of the participants was measured using a light portable digital weighing machine (mini) to the nearest 0.1 kg. The scale was adjusted to a zero level between individual measurements, and height was measured by non-flexible inch tapes to the nearest 0.1 cm while respondents were standing in upright positions.

### Data Quality Control

To control the quality of the data, the questionnaire was translated into the local language (Amharic) and back into English to maintain consistency by experts. A pre-test was done in Bahir Dar to check the questionnaire’s consistency and adaptability on 5% of the total sample size before the actual data collection was started. The data was collected by two health extension workers and two nurses. One nurse and one physiotherapist were supervisors. Two days of intense training were given to data collectors by the principal investigator (KB) and ES on how to approach study participants, how to use the questionnaire and guidelines, and data collection procedures. Furthermore, on each data collection day, the collected data were checked and reviewed for completeness by the main investigator.

### Statistical analysis

The collected data were checked manually for the completeness of relevant information. And the data were checked, coded, and entered into EPI Info version 7 and exported to SPSS version 25 software. Descriptive statistics were done for the frequency of distribution, percentage, frequency, and median. Variables in the study are presented using tables and figures. A binary logistic regression model was performed to identify factors associated with physical inactivity. Assumptions for the binary logistic regression model were checked, like the Hosmer-Lemeshow goodness of fit test (0.41) and multicollinearity. In bivariate analysis, variables with a *p*-value less than 0.25 were entered into multivariable analysis to control for a confounding effect and to examine the association between different independent variables. The result was considered statistically significant in multivariable analysis on the basis of an adjusted odds ratio of 95% and *p*-value less than 0.05.

## Results

### Socio-demographic characteristics of study participants

Out of the total sample size of eight hundred thirty-eight study participants, only 764 were included in this study, with a response rate of 91.2%. Among the total participants, more than a third of fourth 577 (75.5%) were males, and the median age of participants was 66 with a range from 60 to 91 years old. The majority, 550 (72%) of participants were in the 60–69 age range. Four hundred eighty-seven 487 (63.7%) participants were orthodox religion followers, 134 (17.5%) of the participants were not formally educated, 507 (66.4%) were unemployed, and 523 (68.5%) of the participants were married. The details of socio-demographic characteristics are presented in Table 1.

**Table 1:** Socio-demographic characteristics of older adults in Gondar town, North West Ethiopia, 2022 (*N* = 764)

Variables	Categories	Frequency (N)	Percentage (%)
Sex	Male	577	75.5
	Female	187	24.5
Age	60–69	550	72.0
	70–79	126	16.5
	>=80	88	11.5
Religion	Orthodox	487	63.7
	Muslim	196	25.4
	Protestant	59	7.7
	Others	24	3.1
Marital status	Married	523	68.5
	Divorced	44	5.7
	Separated	31	4.1
	Windowed	166	21.7
Occupation	Employed	257	33.6
	Unemployed	507	66.4
Monthly income (ETB)	> 5000	66	8.6
	3001–5000	147	19.2
	1001–3000	418	54.7
	<1000	133	17.4
Education level	College & above	209	27.4
	Secondary school	209	27.4
	Primary school	212	27.7
	No formal educated	134	17.5

ETB = Ethiopian Birr, Others = catholic, Adventist, seven-day followers

### Behavioural and medical conditions of study participants

A total of four hundred fifty-nine participants (60.1%) reported having no prior history of non -communicable diseases (NCDs), 743 (97.3%) participants did not smoke, 757 (99.1%) participants said they did not drink alcohol, 303 (39.7%) participants had poor social support, and a total of 600 (78.5%) of the participants had normal body weight in detail see (Table 2).

**Table 2:** Behavioural and medical conditions of study participant among older adults in Gondar town, Northwest Ethiopia, 2022(*N* = 764)

Variables	Categories	Frequency (N)	Percentage (%)
Having history of NCDs	No	459	60.1
	Yes	305	39.9
Cigarette smoking status	No	743	97.3
	Yes	21	2.7
Alcohol drink	No	757	99.1
	Yes	7	0.9
Social support	Strong social support	170	22.3
	Moderate social support	291	38.1
	Poor social support	303	39.7
Depression	No	427	55.9
	Yes	337	44.1
Body Mass Index	Normal weight	600	78.5
	Under weight	23	3.1
	Over weight	121	15.8
	Obese	20	2.6

NCD = Non communicable disease

### Prevalence of physical inactivity among older adults

The overall prevalence of physical inactivity among older adults in Gondar town was 65.6% (95% CI: 62.1–68.9). The proportion of physical inactivity among significant variables was as follows: 162 (32%) participants were female, 80 (16%) participants were > = 80 years old, 185 (36.9%) participants completed primary education, 126 (25%) participants had no formal education, 448 (89.4%) older adults were unemployed, and 321 (64.1%) had depression symptoms.

### Factors associated with physical inactivity among older adults

All variables were undertaken into bivariable analysis to identify the potential candidates for multivariable logistic regression. Thus, nine variables were found significant at *p*-value < 0.25 in a binary logistic regression analysis. The variables included age, sex, marital status, educational level, occupation, monthly income, body mass index, non-commendable disease, and depression. Then all nine variables were entered into multivariable logistic regression to identify statistically significant independent variables that are predictors of physical inactivity among older adults. Of all independent variables fitted into multivariable logistic regression analysis, only five variables (age, sex, educational level, occupation, and depression) were found to have a significant associated with physical inactivity among older adults. Regarding sex, the odds of likebeing female were about three times higher than those who were males (AOR: 3.053, 95% CI: 1.487–6.267). Older adults who were 80 years of age and older were 4.048 times more likely to be physically inactive (AOR: 4.082, 95% CI: 1.234–13.497) as compared to the 60–69-year-old age group. Unemployed participants had a 10.273 fold increased risk of developing physical inactivity (AOR: 10.273, 95% CI: 5.628–18.753) as compared to employed participants. Similarly, primary school level participants were 3.020 times more likely to be physically inactive (AOR: 3.020, 95% CI: 1.433–6.367) as compared to college and above levels. Older adults who had symptoms of depression were 7.152 times more likely to be physically inactive (AOR: 7.152, 95% CI: 4.786–17.965) as compared to those who had no symptoms of depression (See Table 3).

**Table 3:** Factors associated with physical inactivity among older adults in Gondar town, Northwest Ethiopia, 2022 (*N* = 764)

Variables	Categories	Physical inactivity		COR (95%CI)	AOR (95%CI)
		No	Yes		
Sex	Male	238	339	1	1
	Female	25	162	4.549(2.893–7.153)	**3.053(1.487–6.267)** **
Age	60–69	238	312	1	1
	70–79	17	109	4.891(2.855–8.378)	1.093(0.393–3.043)
	>=80	8	80	7.628(3.617–16.086)	4.082(1.234–13.497) *
Marital status	Married	239	284	1	1
	Divorced	4	40	8.415(2.968–23.860)	2.308(0.465–11.463)
	Separated	5	26	4.376(1.655–11.572)	2.617(0.537–12.755)
	Windowed	15	151	8.472(4.850-14.798)	1.501(0.585–3.842)
Education level	>=College	133	76	1	1
	Secondary	95	114	2.100(1.419–3.107)	1.086(0.558–2.114)
	Primary	27	185	11.991(7.328–19.620)	3.020(1.433–6.367) **
	No formal educates	8	126	27.563(12.785–59.420)	**8.573(2.843–25.847)** **
Occupation	Employed	204	53	1	1
	Unemployed	59	448	29.227(19.470-43.872)	10.273(5.628–18.753)**
Monthly Income	> 5000	52	14	1	1
	3001–5000	98	49	1.857(0.938-3.675)	0.600(0.215–1.672)
	1001–3000	104	314	11.214(5.970-21.065)	0.839(0.302–2.327)
	< 1000	9	124	51.175(20.852-125.595)	1.564(0.356–6.865)
Body Mass Index	Normal	222	378	1	1
	Under wt.	6	17	2.664(0.647–4.283)	0.518(0.103–2.593)
	Over wt.	26	95	2.146(1.349–3.414)	1.170(0.508–2.697)
	Obese	9	11	0.718(0.293-1.759)	0.529(0.110–2.553)
NCDs	No	212	247	1	1
	Yes	51	254	4.275(3.005–6.080)	1.416(0.750–2.673)
Depression	No	247	180	1	1
	Yes	16	321	27.530(12.776–58.410)	7.152(4.786–17.965) **

* Statistically significant at *p* < 0.05, **highly statistically significant at *p* < 0.01, 1 = constant, COR = crude odd ratio, AOR = adjusted odd ratio, CI = confidence interval, NCD = non communicable disease, wt. =weight

## Discussion

This study aimed to determine the prevalence of physical inactivity and its associated factors among older adults in Gondar town, Northwest Ethiopia. The overall prevalence of physical inactivity among the older population in Gondar town, northwest Ethiopia, was 65.6% (95% CI: 62.1–68.9), and age, sex, educational level, occupation, and depression were variables significantly associated with physical inactivity among older adults. The current study finding was consistent with the prevalence of physical inactivity among middle-aged and older adults reported from China (63.1%) [14]. This similarity might be due to the pandemic occurrence of physical inactivity [33]. However, the findings of the current study were lower compared with the study conducted in Brazil (70.1%) [18]. These differences might be related to the lower cutoff criteria of the tool used by the Brazilian study, which is defined as “physical inactivity” as engaging in regular activities that take up fewer than 60 min per week. Nonetheless, the current study’s cut-off points were higher, with regular activities being defined as those that last fewer than 150 min each week.

In addition, the finding of the current study were higher than the study done among adults in Harar town, Ethiopia (45.5%) [23]. This could be due to the fact that the study populations are different, where the study in Harari used adult participants in the age range of 18–64 years old. Evidence also showed that as age increases, physical inactivity increases, resulting in rapid declines in physical activity level, mobility, and functional independence [34]. The present study finding were also higher than studies done in Brazil (50.7%) [13], Malaysia (48.8%) [20], China (24.1%), India (22.0%), and South Africa (50.9%) [35]. This discrepancy might be due to a different physical activity measurement tool and the study participant’s age. For example, the participants’ ages in China, India, and South Africa studies were ≥ 50 years old. Evidence also supported that physical inactivity increased with increasing age, 25.4% among adults aged 50–64 years, 26.9% among those aged 65–74 years, and 35.3% among those aged ≥ 75 years in the USA [36]. An increase in age leads to a decline in physical activity which is attributed loss of muscle mass, physical strength, endurance, flexibility, living a sedentary life, and a loss of functional health [37]. In addition, our study finding is also higher than studies in Malaysia and Brazil due to the short version of the International Physical Activity Questionnaire (IPAQ) to assess physical activity levels where many simple activities were considered as physical activity.

According to the findings of the present study, females were more likely to be physically inactive than males. The finding is supported by the studies done in Ireland [19], China in Shenzhen [14], Malaysia [20], and the USA [36]. The reasons could be due to the fact that females have been affected by various factors, including family and societal roles, physical and psychological issues, and life conditions between men and women [38]. In addition, elderly women are not willing to do physical activity as they are engaged in excess family care, caring for children, and are prone to disease, marriage, and the fear of falling [39]. Thus, women in the current study setting are the main caregivers and home managers in the family, which tends to restrict their participation in physical activities.

The results of this study also showed that people aged 80 and older were four times more likely to be physically inactive than people aged 60 to 69 years. This study is supported by the studies done in Brazil [18] and Malaysia [15]. Evidence also showed increased inactivity in older individuals due to poor health and psychosocial factors such as lack of family support, loneliness, fear of physical injury during exercise, and lack of interest [40]. Another reason could be that the majority of the older age group in this study were retired, which leads to sedentary physical activities.

In addition, the current study finding showed that those who have no formal education and were in primary school were significantly associated with physical inactivity compared to college and those above educational levels. This study’s findings were consistent with the research studies in Brazil [18] and Italy [38]. The reason could be that people with a higher level of education might have a better understanding of the benefits of adequate physical activity and adopt a healthy lifestyle [41].

This study finding also revealed that physical inactivity among unemployed participants were ten times higher compared with employed participants. The finding is also supported by the studies done in Malaysia [15] and low and middle-income countries [35]. These might be unemployed participants who are less likely to perform the recommended level of physical activity that leads to a more sedentary and inactive lifestyle as a result of low socioeconomic status and poor access to resources for participation in physical activity [42, 43].

Furthermore, the present study finding showed that older adults with depression were more likely to be physically inactive as compared to non-depressed older adults. These findings were consistent with the studies done in Italy [38] and Brazil [44]. The possible reason could be that depressed older adults might have diminished interest, pleasure, lack of energy, psychomotor retardation or decreased cognitive function, and lifestyle factors [45].

### Strength and limitation of the study

Our study’s strength was that we assessed physical inactivity using the GPAQ, which has been validated in low- and middle-income countries, to measure physical inactivity and that we adhered to the GPAQ analysis protocol. In addition, we used a large sample size. However, the main limitations of this study were that it did not assess cultural and environmental factors for physical activity, and the data were based on self-reports, which were subjected to recall bias.

## Conclusion

Physical inactivity was relatively high among older adults in Gondar town. Being female, older age, having low levels of education, being unemployed, and having depression symptoms were associated with physical inactivity. We suggest promoting the health benefits of physical activity among females by reducing their burden, older age groups, and unemployed older adults and avoiding depression among individuals.

## Data Availability

The manuscript contains all of the data that is crucial to our findings. Requests for additional information on the dataset and questions about data sharing will be treated in accordance with a reasonable request to uogbelay@gmail.com.

## Abbreviations

AOR: Adjusted Odd Ratio
BMI: Body Mass Index
CI: Confidence Interval
CM: Centimeter
COR: Crude Odd Ratio
ETB: Ethiopian Birr
GDS: Geriatrics Depression Scale
GPAQ: Global Physical Activity Questionnaire
KG: Kilogram
MET: Metabolic Equivalents of Task
NCDs: Non-Communicable Diseases
OSSS: Oslo Social Support Scale
PA: Physical Activity
SPSS: Statistical Package of Social Science
